# Burnout symptoms among physicians and nurses before, during and after COVID-19 care

**DOI:** 10.1590/1518-8345.6820.4047

**Published:** 2023-11-03

**Authors:** Giselle Dayana Valdes-Elizondo, Pablo Álvarez-Maldonado, Maria Angélica Ocampo-Ocampo, Grisel Hernández-Ríos, Arturo Réding-Bernal, Alejandro Hernández-Solís

**Affiliations:** 1 Hospital General de México, Servicio de Neumología, Ciudad de México, México.; 2 Becario del Sistema Nacional de Investigadores, CONACYT, México.

**Keywords:** Burnout, COVID-19, Physicians, Nurses, Pandemics, SARS-CoV-2, Burnout, COVID-19, Médicos, Enfermeras, Pandemias, SARS-CoV-2, Burnout, COVID 19, Médicos, Enfermeiros, Pandemias, SARS-CoV-2

## Abstract

**Objective:**

this study evaluated burnout symptoms among physicians and nurses before, during and after COVID-19 care.

**Method:**

a cross-sectional comparative study in the Pulmonary Care unit of a tertiary-level public hospital. The Maslach Burnout Inventory was used.

**Results:**

280 surveys were distributed across three periods: before (n=80), during (n=105) and after (n=95) COVID-19 care; 172 surveys were returned. The response rates were 57.5%, 64.8% and 61.1%, respectively. The prevalence of severe burnout was 30.4%, 63.2% and 34.5% before, during and after COVID-19 care (p<0.001). Emotional exhaustion (p<0.001) and depersonalization (p=0.002) symptoms were more prevalent among nurses than among physicians. Severe burnout was more prevalent in women, nurses and night shift staff.

**Conclusion:**

the high prevalence of burnout doubled in the first peak of hospital admissions and returned to pre-pandemic levels one month after COVID-19 care ended. Burnout varied by gender, shift and occupation, with nurses among the most vulnerable groups. Focus on early assessment and mitigation strategies are required to support nurses not only during crisis but permanently.

## Abstract (es)

**Objetivo:**

este estudio evaluó síntomas de Burnout entre médicos y enfermeros antes, durante y después de la atención provista a pacientes con la enfermedad COVID-19.

**Método:**

estudio comparativo y transversal realizado en la unidad de Atención Respiratoria de un hospital público de nivel terciario. Se empleó el Inventario de Burnout Maslach.

**Resultados:**

se distribuyeron 280 encuestas entre los tres períodos: antes (n=80), durante (n=105) y después (n=95) de la atención a pacientes con COVID-19; se obtuvieron 172 encuestas respondidas. Las tasas de respuesta fueron 57,5%, 64,8% y 61,1%, respectivamente. Los valores de prevalencia de Burnout grave fueron 30,4%, 63,2% y 34,5% antes, durante y después de la atención a pacientes por la enfermedad del coronavirus 2019 (p<0,001). Los síntomas de agotamiento emocional (p<0,001) y despersonalización (p=0,002) fueron más prevalentes entre los enfermeros que entre los médicos. El Síndrome de Burnout grave fue más prevalente en las mujeres, los enfermeros y el personal del turno noche.

**Conclusión:**

la elevada prevalencia de Burnout se duplicó en el primer pico de internaciones y regresó a niveles previos a la pandemia un mes después de finalizada la atención a pacientes por la enfermedad del coronavirus 2019. El Síndrome de Burnout varió por sexo, turno de trabajo y ocupación, y los enfermeros representaron los grupos más vulnerables. Es necesario enfocarse en estrategias de evaluación y mitigación tempranas para asistir a los enfermeros, no solo durante la crisis sino permanentemente.

## Abstract (pt)

**Objetivo:**

este estudo avaliou os sintomas de burnout entre médicos e enfermeiros antes, durante e após o cuidado dos pacientes contaminados com o COVID-19.

**Método:**

estudo transversal comparativo realizado na unidade de Atenção Pulmonar de um hospital público de nível terciário. Foi utilizado o Inventário de Burnout de Maslach.

**Resultados:**

280 formulários de pesquisa foram distribuídos em três períodos: antes (n=80), durante (n=105) e após (n=95) os cuidados dos pacientes contaminados com COVID-19; 172 formulários foram respondidos. As taxas de resposta foram de 57,5%, 64,8% e 61,1%, respectivamente. A prevalência de burnout grave foi de 30,4%, 63,2% e 34,5% antes, durante e após o atendimento dos pacientes (p<0,001). Os sintomas de exaustão emocional (p<0,001) e despersonalização (p=0,002) foram mais prevalentes entre os enfermeiros do que entre os médicos. O burnout grave foi mais prevalente em mulheres, enfermeiros e funcionários do turno da noite.

**Conclusão:**

a alta prevalência de burnout dobrou no primeiro pico de internações hospitalares e voltou aos níveis pré-pandemia um mês após o término dos cuidados dos pacientes contaminados com COVID-19. O burnout variou de acordo com o sexo, turno e profissão, encontrando-se os enfermeiros entre os grupos mais vulneráveis. O foco na avaliação precoce e nas estratégias de mitigação é necessário para apoiar os enfermeiros não apenas durante a crise, mas de forma permanente.

**Highlights:**

**(1)** Several manifestations of psychological stress have been reported in COVID-19 care.

**(2)** This study shows high prevalence of burnout in Mexican healthcare personnel.

**(3)** Women, nurses and night shift staff were more prone to burnout.

**(4)** Burnout returned to pre-pandemic levels after the end of COVID-19 care.

## Introduction

According to the World Health Organization, burnout is an “occupational syndrome characterized by energy depletion or exhaustion, increased mental distance from one’s job, negativism or cynicism related to it, and reduced professional efficacy resulting from chronic workplace stress” ^(^ 1 ^)^. Healthcare personnel is considered a vulnerable population due to its propensity to experience different forms of work-related stress ^(^ 2 ^)^. Since the beginning of the SARS-CoV-2 pandemic, healthcare personnel faced challenges in caring for patients with the coronavirus disease 2019 (COVID-19). Increased workload; lack of space, equipment and supplies; fear of contagion; and social stigma have all contributed to physical and mental health problems among physicians and nurses at the front line of the efforts to combat COVID-19 ^(^ 3 ^-^ 5 ^)^. 

Severe burnout among healthcare staff members, likely under-reported or not reported at all but showing rates as high as 60% before the pandemic ^(^ 6 ^)^, has been found to be higher during population contagion peaks ^(^ 7 ^)^. Burnout is known to negatively affect healthcare quality due to poor staff performance and increasing propensity to medical errors ^(^ 2 ^)^. During the pandemic, information on mental health outcomes over time has been extensively described, but provides no data on the tested sample mental health before the outbreak ^(^ 8 ^)^. This study aimed at evaluating burnout symptoms in healthcare personnel from a Pulmonary Care unit in Mexico City before care, during care and one month after cessation of the care provided to hospitalized patients with COVID-19, as well at examining whether the symptoms differed between physicians and nurses. 

## Method

### Study design and setting

The Strengthening the Reporting of Observational Studies in Epidemiology (STROBE) reporting guidelines were used ^(^ 9 ^)^. This cross-sectional study was conducted in the Pulmonary Care unit of a tertiary-level public teaching hospital in Mexico City, Mexico, which had been temporarily converted to care for COVID-19 patients. The Pulmonary Care unit’s facilities include a 60-bed hospitalization floor, a 12-bed Intensive Care Unit, operating rooms, an eight-bed Emergency Room, and outpatient consultation rooms. 

### Period, population, selection criteria and definition of the sample

Physicians and nurses who were permanently employed were surveyed one week before admission of the first COVID-19 patient (March 16 ^th^, 2020, denoted as the “PRE” period), during the first peak of hospital admissions in Mexico (July 2 ^nd^, 2020, denoted as the “PEAK” period), and 1 month after the unit was deconverted and the staff had returned to their normal duties (July 4 ^th^, 2021, denoted as the “POST” period). Once the Pulmonary Care unit was deconverted, its staff did not return to treat COVID-19 patients, at least not until the last survey was applied. Temporary staff took care of COVID-19 patients admitted to the hospital in another redesigned area. 

### Data collection and instruments

The surveys were anonymous and, to avoid negatively affecting the response rate, personal information was requested only regarding the type of staff, gender, working schedule and a second job in COVID-19 areas at another hospital. The researchers personally delivered the surveys to each respondent on the scheduled days during the different shifts or, if necessary, on the 2 days after the scheduled day for night and weekend shifts. The surveys were returned to a mailbox in the Pulmonary Care unit’s administrative office. After distribution of the surveys, the mailbox remained in place for 48 hours for the morning and afternoon shifts, and for 76 hours for the night shift during all three data collection periods. Other healthcare workers in the Pulmonary Care unit, including administrative personnel, cleaning staff, respiratory therapists and stretcher bearers were not surveyed owing to the small number of employees in each category and their permanent turnover in other (non-COVID-19) hospital areas.

The healthcare personnel was evaluated with the Maslach Burnout Inventory-Human Services Survey (MBI-HSS), validated for the Mexican population in 2016 ^(^ 10 ^)^ and containing 22 items divided into three subscales: Emotional Exhaustion (EE, nine items), Depersonalization (DP, five items), and Personal Accomplishment (PA, eight items). The total scores for the three dimensions are 54, 30 and 48, respectively. Burnout was considered high with EE scores ≥27, DP scores ≥13 or PA scores ≤31; moderate with EE between 17 and 26, DP between 7 and 12 or PA between 32 and 38; and low with EE ≤16, DP ≤6 or PA ≥39. The overall burnout level was considered high if any of the three subscales were rated high. 

The following measures were implemented in our unit to mitigate and manage stress from the beginning of the pandemic: 1) Provision of a second day off for nurses every 2 weeks, and flexible schedules for physicians, 2) Allowing music to be played in Nursing stations during daytime hours, according to the staff preferences, 3) Adoption of an open space in the same building so that the staff did not have to go to the hospital dining room to have meals, and 4) Provision of personal assistance by psychologists in the same building, who were present during the day and on call at night. According to governmental mandate, healthcare workers with one or more comorbidities, such as diabetes, hypertension or cancer, and/or those over 60 years of age, were sent home during the pandemic. These measures became a routine carried out throughout most of the pandemic in the Pulmonary Care unit and were allowed from the beginning until the last data collection during the POST period. Additional days off, flexible schedules and mental health assistance remained in force until December 31 ^st^, 2022. Music in Nursing stations had no longer been allowed since the unit was deconverted, and the dining areas within the building remain until the present day as an alternative to the hospital’s main dining room. 

### Data treatment and analysis

The chi-square test and Fisher’s exact test were used to evaluate differences in frequencies. The estimated marginal mean of the three dimensions from MBI-HSS was also compared according to type of occupation and shift. The Statistical Package for the Social Sciences (SPSS ^®^) software, v.25.0, was used for statistical analysis. 

### Ethical aspects

Permission to conduct the study was obtained from our Institutional Review Boards’ ethics and research committees and the research complies with the provisions set forth in the Declaration of Helsinki, as revised in Edinburgh in 2000.

## Results

A total of 280 surveys were distributed across the three periods (PRE=80, PEAK=105, POST=95). The variations in the total number of staff members were due to hiring, absenteeism and termination. The Pulmonary Care unit started care for COVID-19 patients with 80 workers, of which 25 correspond to physicians and 55 to nurses, subsequently sending home those with chronic diseases and aged over 60 years old. The proportion of women that received a survey during each period was 61.3%, 65.7% and 68.4% for the PRE, PEAK and POST periods, respectively.

One hundred and seventy-two surveys were returned (62.2% were answered by women): 46 for the PRE period, 68 for the PEAK period and 58 for the POST period. The response rates were 57.5% (46/80), 64.8% (68/105) and 61.1% (58/95), respectively. The data on the respondents’ gender, occupation and work shift are detailed in Table 1. From the PRE period to the PEAK period, one physician abandoned his position (1.3% dropout rate), and four physicians and 27 nurses were hired. From the PEAK period to the POST period, one physician and nine nurses resigned (9.5% dropout rate), and all of them were personnel recently hired during the PEAK period. Of the total number of respondents in each period, the percentages with a second job in COVID-19 care at another hospital were 10.8% in the PRE period, 8.8% in the PEAK period and 8.6% in the POST period, and all were physicians. The prevalence of burnout was 30.4% during the PRE period, 63.2% during the PEAK period, and 34.5% during the POST period (p<0.001). Among the physicians, no gender differences were observed; however, women predominated among the nurses (84%, p=0.008; Table 1). 

During the PEAK period, the nurses presented more burnout symptoms than the physicians, with statistically significant increased values in the Emotional Exhaustion (p<0.001) and Depersonalization (p=0.002) subscales ( Table 2). 

**Figure 1 -:**
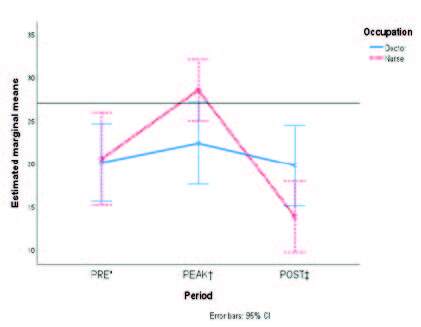

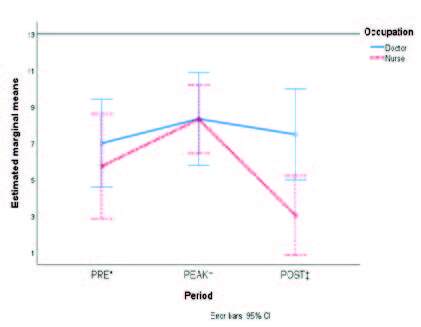

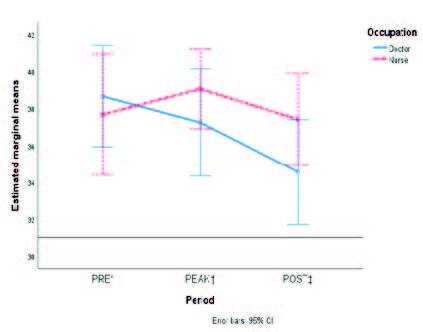
The estimated marginal means of each of the three variables from the Maslach Burnout Inventory are compared in the three periods according to type of occupation: A) Emotional Exhaustion; B) Depersonalization; and C) Personal Accomplishment

Figure 1 shows the marginal means of the accrued scores of each dimension from MBI-HSS according to type of staff. Although certain overlap of the standard error bars is noticed, differences in the burnout symptoms occurred in the POST phase rather than any other when comparing physicians to nurses. 

**Table 1 -:** Baseline characteristics of the study population according to occupation and data collection period. Mexico City, Mexico, 2020-2021

	**Occupation**							
**Variables**	**Physicians (n = 76)**				**Nurses (n = 96)**			
	PRE *	PEAK †	POST ‡	p-value	PRE *	PEAK †	POST ‡	p-value
	(n = 27)	(n = 24)	(n = 25)		(n = 19)	(n = 44)	(n = 33)	
Gender, n (%)								
Female	9 (33.3)	7 (29.2)	10 (40.0)		19 (100.0)	32 (72.7)	30 (90.9)	
Male	18 (66.7)	17 (70.8)	15 (60.0)	0.722 §	0 (0.0)	12 (27.3)	3 (9.1)	0.008 ^ǁ^
Work schedule, n (%)								
Day	17 (63.0)	16 (66.7)	14 (56.0)		12 (63.2)	40 (90.9)	24 (72.7)	
Night	10 (37.0)	8 (33.3)	11 (44.0)	0.736 §	7 (36.8)	4 (9.1)	9 (27.3)	0.020 ^ǁ^

* PRE = It denotes the pre-pandemic period;

† PEAK = It denotes the first peak of COVID-19 hospital admissions in Mexico;

‡ POST = It denotes the period after COVID-19 care ended;

§ Frequency differences were compared with the chi-square test;

ǁǁ Frequency differences were compared with Fisher’s exact test

**Table 2 -:** Maslach Burnout Inventory subscale categorization of the study population according to occupation and data collection period. Mexico City, Mexico, 2020-2021

	**Occupation**							
**Variables**	**Physicians (n = 76)**				**Nurses (n = 96)**			
	PRE *	PEAK †	POST ‡	p-value	PRE *	PEAK †	POST ‡	p-value
	(n = 27)	(n = 24)	(n = 25)		(n = 19)	(n = 44)	(n = 33)	
Emotional exhaustion, n (%)								
Low	14 (51.9)	9 (37.5)	13 (52.0)		7 (36.8)	6 (13.6)	22 (66.7)	
Moderate	6 (22.2)	7 (29.2)	2 (8.0)		4 (21.1)	13 (29.5)	9 (27.3)	
High	7 (25.9)	8 (33.3)	10 (40.0)	0.342 ^ǁ^	8 (42.1)	25 (56.8)	2 (6.1)	<0.001 ^ǁ^
Depersonalization, n (%)								
Low	16 (59.3)	11 (45.8)	14 (56.0)		11 (57.9)	25 (56.8)	29 (87.9)	
Moderate	5 (18.5)	6 (25.0)	5 (20.0)		8 (42.1)	10 (22.7)	3 (9.1)	
High	6 (22.2)	7 (29.2)	6 (24.0)	0.912 §	0 (0.0)	9 (20.5)	1 (3.0)	0.002 ^ǁ^
Personal accomplishment, n (%)								
Low	16 (59.3)	13 (54.2)	7 (28.0)		8 (42.1)	27 (61.4)	19 (57.6)	
Moderate	7 (25.9)	5 (20.8)	10 (40.0)		9 (47.4)	12 (27.3)	9 (27.3)	
High	4 (14.8)	6 (25.0)	8 (32.0)	0.174 §	2 (10.5)	5 (11.4)	5 (15.2)	0.553 ^ǁ^

* PRE = It denotes the pre-pandemic period;

† PEAK = It denotes the first peak of COVID-19 hospital admissions in Mexico;

‡ POST = It denotes the period after COVID-19 care ended;

§ Frequency differences were compared with the chi-square test;

ǁǁ Frequency differences were compared with Fisher’s exact test

*PRE = It denotes the pre-pandemic period; †PEAK = It denotes the first peak of COVID-19 hospital admissions in Mexico; ‡POST = It denotes the period after COVID-19 care ended

**Figure 2 -:**
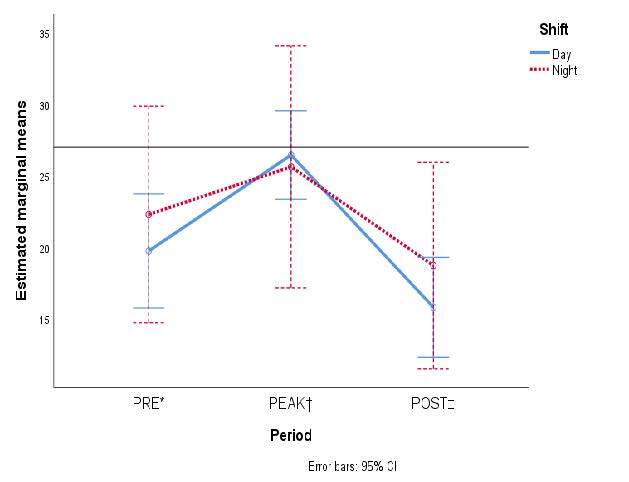

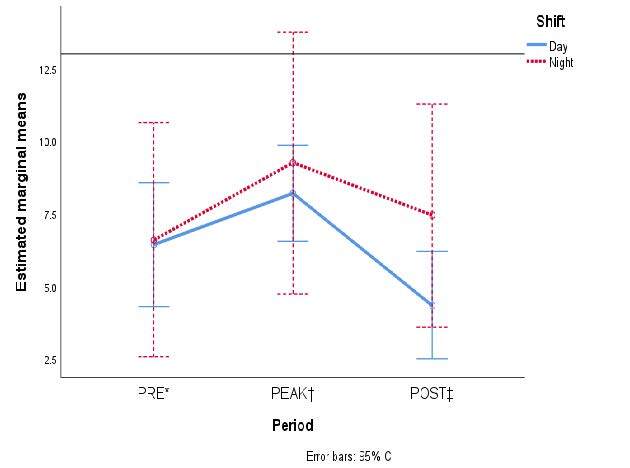

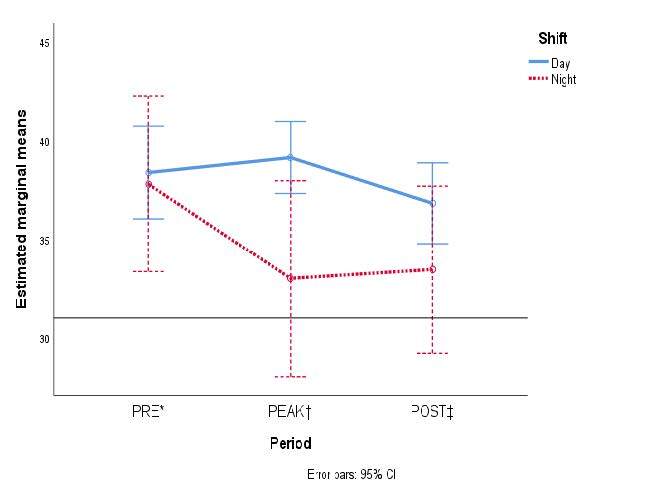
The estimated marginal means of each of the three variables from the Maslach Burnout Inventory are compared in the three periods according to healthcare personnel shifts: A) Emotional Exhaustion; B) Depersonalization; and C) Personal Accomplishment

Burnout was more common among night shift staff members, and the Personal Accomplishment subscale was more affected than in the day shift staff. Figure 2 shows the marginal means of the accrued scores of each dimension from MBI-HSS according to work schedule. 

*PRE = It denotes the pre-pandemic period; †PEAK = It denotes the first peak of COVID-19 hospital admissions in Mexico; ‡POST = It denotes the period after COVID-19 care ended

## Discussion

Burnout syndrome refers to work-related stress, which affects a worker’s personality and self-esteem. We found that 30.4% of the healthcare personnel in our unit experienced severe burnout before the start of COVID-19 care, and the proportions were similar between physicians and nurses. The prevalence of burnout doubled (63.2%) during the PEAK period, a prevalence value in agreement with findings from other countries during the same epidemic-curve period (China: 69.7%; United Kingdom: 55%) ^(^ 11 ^-^ 12 ^)^, and returned to pre-pandemic levels (34.5%) in the POST period. The reasons that explain the drop in half in burnout a month after closing the Pulmonary Care unit for COVID-19 care are not clear. A simple explanation would be that the stressor was removed ^(^ 5 ^)^. During the PEAK period, our staff experienced high pressure, performance under uncertainty, lack of available vaccines and fear of being infected and infecting family members. As in other public hospitals in Mexico, in our unit vacations were postponed, an aspect we believe to have further decreased enthusiasm for work. The return to normal duties had to exert some impact on burnout reduction in our healthcare personnel; nevertheless, the pandemic was still going on during the POST period and isolation measures were permanent inside and outside hospitals, as well as the risk of becoming infected. The time elapsed from the first peak of contagions in Mexico to the POST period (one calendar year) should have generated resilience, as well as having vaccines and accumulated experience in patient management could have generated skill-based adaptive characteristics, reducing fear in the healthcare personnel ^(^ 13 ^)^. 

Using a short version of MBI-HSS, a study conducted in a hospital belonging to the Mexican Social Security Institute that converted to COVID-19 care, found small variations in the burnout levels of front-line staff before, during and after the first peak of hospital admissions ^(^ 7 ^)^. According to the same group of researchers, the stress levels, a key factor affecting burnout, were lower during the second peak than in the first peak of hospital admissions ^(^ 14 ^)^. 

In April 2020, in conjunction with the Mexican Psychiatric Association, the Mexican Ministry of Health launched a program to address the mental health needs of front-line healthcare personnel ^(^ 15 ^)^; however, despite its accessibility and scope, the service did not receive substantial demand, possibly because of the normalization of stress or fears of social stigma. In our unit, despite support from mental health specialists in the building, the demand for mental health care was also low. 

According to a study, the burnout levels due to depersonalization were lower in México than in other Latin American countries ^(^ 16 ^)^. Far above physicians, nurses showed the highest percentage of emotional exhaustion among United States of America hospitals in 2021 to 2022 ^(^ 17 ^)^. Nurses caring for COVID-19 patients in a Pakistani population reported low personal accomplishment above emotional exhaustion and depersonalization ^(^ 18 ^)^. In our study, personal accomplishment increased during the PEAK period in Mexican nurses but not among physicians. A recent meta-analysis found comparable levels of overall burnout prevalence and prevalence by subscale between physicians and nurses when pooling observational studies from around the world ^(^ 19 ^)^. 

Several psychological stress manifestations have been reported in women during the pandemic ^(^ 20 ^)^, including frequent crying and suicidal ideation ^(^ 21 ^)^. Pre-existing anxiety among Mexican female healthcare workers has been associated with acute stress during the first peak of hospital admissions; in addition, young women have been found to have lower resilience and more pre-existing psychological symptoms, thus leading to more post-traumatic stress ^(^ 7 ^)^. Immediately before the pandemic, high prevalence of moral distress among nurses from a University Hospital in Brazil was associated to low personal accomplishment and high emotional exhaustion, with those who were not permanently employed and had a morning shift schedule as the most affected ^(^ 22 ^)^. Pre-existing anxiety predict burnout symptoms of emotional exhaustion and depersonalization according to a study conducted with Spanish female nurses ^(^ 23 ^)^. Women make up most of the Nursing personnel worldwide, and working in this profession has been reported as an independently associated factor to develop burnout ^(^ 3 ^)^. There has been substantial discrimination, abuse and even sexual harassment against women in Neurosurgery programs in Mexico and other Latin American countries during the pandemic, resulting in high burnout levels due to emotional exhaustion (44%) and depersonalization (39%), although without markedly affecting personal accomplishment ^(^ 16 ^)^. These findings are consistent with those from studies conducted at the beginning of the pandemic, in which the Personal Accomplishment dimension was not significantly affected ^(^ 4 ^,^ 24 ^)^. Given that 84% of the surveyed nurses were women, the factors described above might have contributed to the differences in burnout prevalence between physicians and nurses. Another stress factor affecting female nurses providing front-line care in Mexico is that most of them are also heads of households who care for children and older relatives, who were oftentimes forced to leave their homes and live in specially designed shelters for fear of infecting their families. Ensuring adequate staffing levels and resources is critical to lowering burnout among nurses during a crisis ^(^ 25 ^)^; nevertheless, financial incentives and alternatives for continual and effective communication between healthcare workers and their families during their shifts (e.g., video calls and Internet coverage) might potentially ease this burden ^(^ 26 ^-^ 28 ^)^. 

Our results are limited by the single-center nature of the study and by non-consideration of other factors related to the development of burnout. Non-respondents might have been affected by stress and burnout. We tried to keep the survey simple, and we did not collect personal or redundant data to avoid discouraging stressed staff from answering. Even though the response rate was acceptable in our study, bias may still remain if burnout prevalence and severity among the non-respondents were not similar to those of the respondents. Unfortunately, information was not available on non-respondents.

The main study strength is that we were able to apply the instrument longitudinally at the three moments: during, after and, especially, before the care provided to COVID-19 patients. To the best of our knowledge, no study has described data on the stress of the same healthcare personnel in the pre-COVID-19 period and its subsequent evolution.

## Conclusion

We conclude that the prevalence of burnout symptoms in healthcare personnel was high before the pandemic, doubled at the first peak of hospital admissions in Mexico, and returned to pre-pandemic levels one month after the end of COVID-19 care. During the pandemic, burnout varied by gender, shift and occupation among healthcare staff members. As nurses appear to be one of the most vulnerable groups to burnout, early assessment and rapid implementation of actions to mitigate psychological stress among them are required, not only during a crisis but permanently.
